# A Nomogram Prediction Model and Scoring System for Resistance in Acute Myeloid Leukemia Patients Treated with Venetoclax Combined with Hypomethylating Agents

**DOI:** 10.3390/curroncol33060357

**Published:** 2026-06-13

**Authors:** Qingqing Fan, Yujiao Guo, Xiang Hui, Yu Zhang, Jianrui Li, Jinhua Liang, Yongqing Wang

**Affiliations:** 1 Department of Pharmacy, The First Affiliated Hospital with Nanjing Medical University, Nanjing 210029, China; 2 Department of Hematology, The First Affiliated Hospital with Nanjing Medical University, Nanjing 210029, China; 3 Research Division of Clinical Pharmacology, The First Affiliated Hospital with Nanjing Medical University, Nanjing 210029, China; 4 School of Pharmacy, Nanjing Medical University, Nanjing 211166, China

**Keywords:** venetoclax, BCL2, drug resistance, acute myeloid leukemia, prediction model

## Abstract

To investigate the predictive factors for resistance to VEN combined with HMAs in the treatment of AML, construct a drug resistance prediction model, and visualize the model. A retrospective analysis was conducted on 74 AML patients. Multivariate logistic regression was used to identify independent predictors of primary resistance, based on which a nomogram model and a risk scoring system for drug resistance were constructed. The results showed that KIT (*p* = 0.012), TP53 (*p* = 0.010), and FAB-M5 (*p* = 0.059) were significantly associated with primary resistance to VEN. A nomogram prediction model incorporating FAB-M5, KIT, and TP53 was established. Based on the nomogram model, a drug resistance prediction scoring tool comprising three variables was developed, categorizing patients into high-risk (6–10 points), intermediate-risk (3–5 points), and low-risk (0–2 points) groups. Significant differences in NR rates were observed among the three risk groups (*p* < 0.001). KIT, TP53, and FAB-M5 are independent factors influencing VEN resistance. The constructed nomogram prediction model and scoring system may provide valuable references for predicting primary resistance to VEN.

## Simple Summary

Acute myeloid leukemia (AML) is a type of blood cancer. In this study, we looked at patients with AML who were treated with a combination of two medicines (called VEN and HMAs). We wanted to find out which factors might predict that the cancer does not respond to these medicines. We studied 74 patients and analyzed their medical data. We found that three specific features—certain changes in two genes (KIT and TP53) and a specific subtype of AML (called FAB-M5)—were linked to a higher chance of the medicines not working. Using this information, we created a simple scoring tool that divides patients into low, medium, or high risk for treatment resistance. This tool may help doctors predict whether a patient is likely to benefit from this medicine combination, allowing for better treatment decisions in the future.

## 1. Introduction

Acute myeloid leukemia (AML) is an aggressive hematologic malignancy of the hematopoietic system. The median age at AML diagnosis is 68 years, and the incidence among elderly patients has risen sharply in recent years [1]. The primary therapeutic strategies for AML patients have traditionally included intensive induction chemotherapy, consolidation therapy, and allogeneic hematopoietic stem cell transplantation. However, elderly patients are often not optimal candidates for conventional cytotoxic induction therapy and typically receive lower-intensity chemotherapy regimens based on hypomethylating agents (HMAs), such as decitabine (DAC) and azacitidine (AZA). Currently, the response rate to HMA monotherapy is approximately 10–50%, but the time to best response is prolonged (3.5–4.3 months), with a median overall survival of less than one year [2,3,4].

Venetoclax (VEN) is a first-in-class, orally bioavailable small-molecule inhibitor that selectively targets B-cell lymphoma-2 (BCL2), an anti-apoptotic protein overexpressed in many hematologic malignancies. The U.S. Food and Drug Administration (FDA) approved VEN in 2016 for use in combination with AZA in adults with newly diagnosed AML who are 75 years of age or older, or who have comorbidities that preclude intensive induction chemotherapy. This approval marked a paradigm shift in the management of elderly or unfit AML patients, a population historically associated with poor outcomes due to treatment-related toxicity and chemoresistance. The combination of VEN with HMA such as AZA or DAC, or with low-dose cytarabine, has emerged as a highly effective, lower-intensity regimen. Pivotal phase 3 trials, notably the VIALE-A study, demonstrated impressive efficacy: a median overall survival (OS) of 14.7 months versus 9.6 months with AZA alone, and a composite complete remission (CR/CRi) rate of 66–71% [5]. Real-world data have largely corroborated these results, showing rapid achievement of deep responses, often within the first or second cycle, and improved quality of life compared with conventional care [6,7,8,9]. Despite these advances, primary and acquired resistance to VEN-based therapy remains a major clinical challenge. Approximately 30–40% of patients exhibit primary refractoriness, while many responders eventually relapse after a median duration of response of 6–12 months [10,11].

Current research on VEN resistance in AML remains predominantly anchored in basic science investigations, with a relative scarcity of robust, large-scale clinical studies. While preclinical models have elucidated multiple molecular mechanisms, such as BCL2 mutations, MCL-1 overexpression, and the emergence of TP53 mutations, translational data directly linking these findings to patient outcomes are still limited [11,12,13,14]. This imbalance highlights an urgent need to bridge laboratory discoveries with bedside applications. Given the high cost of VEN-based regimens, identifying reliable factors that influence the development of primary or acquired resistance has become crucial for optimizing therapeutic efficacy, improving cost-effectiveness, and sparing patients from ineffective therapy and its associated toxicities.

In this context, predictive modeling tools such as nomograms have gained increasing attention as practical solutions for personalized medicine. A nomogram model integrates multiple independent predictive variables—including clinical parameters (age, cytogenetic risk), molecular markers (FLT3-ITD, TP53 status)—into a single, user-friendly visual tool. By generating a graphical representation of individualized probabilities, nomograms translate complex statistical models into clinically actionable scores that clinicians can calculate quickly during patient consultations. Nomograms enable efficient, precise individualized risk assessment. As more real-world data and prospective studies accumulate, the refinement and widespread adoption of such nomogram models are expected to play a pivotal role in overcoming VEN resistance challenges and maximizing clinical benefit for elderly or unfit AML patients [15,16].

Based on this background, this study aims to evaluate the efficacy and safety of VEN in combination with HMAs for the treatment of AML through a retrospective cohort study. We systematically collect clinical and molecular factors potentially associated with the development of primary resistance to VEN-based therapy, including patient demographics, cytogenetic risk classification, prior treatment, and mutation profiles of genes. Multivariable logistic regression analysis will be performed to identify independent predictors of VEN resistance. Based on these identified factors, we will construct and internally validate a nomogram model for predicting the individual risk of primary VEN resistance before treatment initiation. The predictive results will be presented graphically using a user-friendly scoring system that translates each patient’s unique risk profile into an intuitive probability score. This model is intended to provide individualized and visual risk assessment for AML patients undergoing VEN plus HMA therapy, thereby supporting early identification of patients unlikely to respond to standard doublet therapy.

## 2. Methods

### 2.1. Study Population and Treatment Regimen

Patients diagnosed with AML at Jiangsu Province Hospital between 1 July 2023 and 30 November 2025 were retrospectively enrolled, including those with myelodysplastic syndrome-related secondary AML (MDS-sAML). This study was approved by the Ethics Committee of Jiangsu Province Hospital (approval code: 2025-SR-497; approval date: 17 June 2025). Inclusion criteria: age ≥ 18 years; receipt of at least one treatment cycle combining VEN and HMAs, and availability of complete response evaluation. Exclusion criteria: patients who received less than one full treatment cycle due to death, treatment discontinuation, regimen change, or other reasons. Treatment regimen: VEN was administered at 100 mg on day 1, 200 mg on day 2, and 400 mg on days 3–28, with dose adjustments based on bone marrow suppression and drug interactions (e.g., concomitant use of azole antifungals for prophylaxis). AZA was given at a dose of 75 mg/m^2^ on days 1–7.

### 2.2. Data Collection

Data were collected by reviewing the electronic medical record system and included the following information for enrolled patients: demographic characteristics, AML subtype (newly diagnosed, non-newly diagnosed, MDS-sAML), French-American-British (FAB) classification, molecular and cytogenetic profiles, risk stratification, therapeutic and concomitant medications, VEN trough concentration (C0, ng/mL), treatment response evaluation, occurrence of adverse events, and clinical outcomes. AML subtypes and disease risk were classified according to the 2016 World Health Organization classification and the 2022 European LeukemiaNet (ELN) risk stratification, respectively.

### 2.3. Evaluation Criteria and Definitions

Treatment response evaluation: (1) Treatment response was defined according to the ELN criteria. Evaluated endpoints included: complete remission (CR), complete remission with incomplete hematologic recovery (CRi), morphologic leukemia-free state (MLFS), partial remission (PR), and non-remission (NR). The composite complete remission rate (CRc) was defined as CR + CRi. (2) The overall response rate (ORR) was defined as the proportion of patients achieving CR, CRi, or PR after treatment.

Survival Evaluation: Overall survival (OS) was calculated from the initiation of VEN-based therapy until death from any cause or the last follow-up (censored at the time of loss to follow-up). Event-free survival (EFS) was defined as the time from the start of VEN combination therapy until disease progression, relapse, death from any cause, or the last follow-up.

Primary Resistance: Primary resistance was defined as failure to achieve any treatment response within the ORR categories (i.e., NR) after receiving at least one cycle of VEN-based therapy. This was informed by Dhakal et al. [17], who noted that bone marrow blast decline often occurs within the first 1–2 cycles. Although stricter than ELN 2022 criteria, this definition better aligns with the rapid response kinetics of VEN regimens.

Safety Evaluation: Adverse events (AEs) were assessed primarily according to the Common Terminology Criteria for Adverse Events (CTCAE) v5.0 issued by the U.S. National Cancer Institute.

### 2.4. Statistical Analysis

Continuous variables conforming to a normal distribution are presented as mean ± SD and were compared between groups using the *t*-test or analysis of variance (ANOVA). Continuous variables not conforming to a normal distribution are presented as median (IQR) and were compared between groups using non-parametric tests. Categorical variables are presented as number (percentage) and were compared using the chi-square test or Fisher’s exact test.

Little’s MCAR test was employed to assess the missing data mechanism. For data missing not at random, multiple imputation was used to handle missing values. Binary logistic regression was applied to analyze the association between VEN blood concentration and resistance.

Survival curves were plotted using the Kaplan–Meier method, and survival rates were calculated. Cox proportional hazards regression model was employed for survival analysis to identify independent factors affecting survival. Results are expressed as hazard ratios (HR) with their 95% confidence intervals (CIs).

Statistical analyses were performed using SPSS (v27.0) and R (v4.5.2). A two-sided *p*-value of <0.05 was considered statistically significant.

### 2.5. Construction of a Nomogram Prediction Model and a Predictive Scoring System

Univariable analysis was performed to screen variables with a *p*-value < 0.2 for inclusion in the subsequent multivariable Logistic regression analysis. Based on the model established through multivariable Logistic regression, a nomogram for predicting VEN resistance was constructed.

A simplified scoring system was developed using the β coefficients of the independent factors from the final model as reference values. Weights for other variables were calculated based on these references. The scoring system was formulated by summing the weighted scores assigned to each variable.

## 3. Results

### 3.1. Patients’ Characteristics

A total of 74 AML patients were enrolled in this study, including 44 males and 30 females, with a mean age of 64.18 ± 13.03, 65 (55, 74) years. Among them, 62 cases (83.8%) were newly diagnosed AML, 8 cases (10.8%) were AML transformed from MDS, and 12 cases (16.2%) were relapsed/refractory AML. According to the 2022 ELN risk stratification, there were 12 (16.2%), 14 (18.9%), and 42 (56.6%) patients in the favorable, intermediate, and adverse-risk categories, respectively (6 patients were not assigned to any group because there was insufficient information to perform risk stratification). The top five most frequently mutated genes included FLT3-ITD (22.97%), DNMT3A (17.57%), NPM1 (16.22%), ASXL1 (16.22%), WT1 (10.81%), TP53 (10.81%), NRAS (10.81%), IDH2 (9.46%). The characteristics of the enrolled patients are presented in Table 1.

### 3.2. The Impact of VEN Blood Concentration on Resistance

There were 21 missing cases for the VEN C0 variable in the original data (missing rate 28.4%, n = 53). Evaluation of the missing data mechanism indicated that the data was not missing completely at random (χ^2^ = 8.585, df = 3, *p* = 0.035). Following multiple imputation (m = 5, handling 28.4% missing data), the pooled mean for C0 was 2648.01 ng/mL (95% CI: 2217.41–3078.61). Compared to the original data (2569.56 ± 1740.88, n = 53), the precision of the post-imputation estimate improved, while accounting for the uncertainty introduced by the imputation process (relative variance increase = 27.4%).

The results from the pooled binary logistic regression analysis after multiple imputation showed no statistically significant association between VEN C0 and the risk of developing resistance (OR = 1.000, 95% CI: 1.000–1.001, *p* = 0.152). This suggests that, within the current study sample, a one-unit increase in VEN blood concentration did not significantly alter the risk of developing resistance. Evaluation of the imputation model quality showed that the relative efficiency met statistical requirements.

### 3.3. Risk Factors Associated with VEN Primary Resistance

Results from the multivariate logistic regression analysis indicated that the incidence of primary resistance was significantly higher in patients with KIT mutations compared to those without KIT mutations (75.0% vs. 15.7%, *p* = 0.012), and similarly higher in patients with TP53 mutations compared to those without TP53 mutations (50.0% vs. 15.2%, *p* = 0.010). Both KIT and TP53 were identified as independent factors influencing primary resistance to VEN combined with HMAs therapy. Furthermore, the incidence of primary resistance was significantly higher in the FAB-M5 subgroup than in the non-FAB-M5 subgroup (37.5% vs. 16.67%, *p* = 0.059), suggesting an association between the FAB-M5 subtype and VEN primary resistance.

### 3.4. Model Development

**Figure 1:**
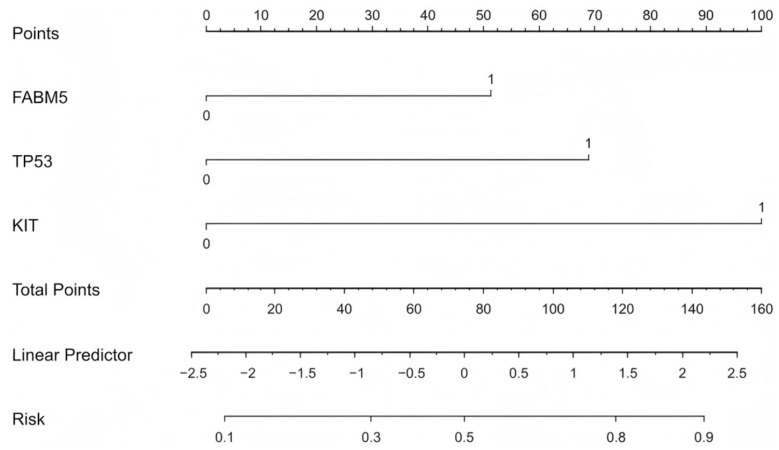
Nomogram for Predicting VEN Primary Resistance.

Variables with a *p*-value < 0.2 were screened by univariable analysis, which included FAB-M5 subtype (*p* = 0.103), KIT mutation (*p* = 0.020), TET2 mutation (*p* = 0.138), and TP53 mutation (*p* = 0.058). The variables identified from the univariable analysis were then entered into univariable logistic regression analysis, resulting in the selection of three variables. These three variables were subsequently included in a multivariable logistic regression analysis. The results demonstrated that KIT mutation (*p* = 0.012), TP53 mutation (*p* = 0.010), and the FAB-M5 subtype (*p* = 0.059) were independent factors influencing VEN resistance, as presented in Table 2. Finally, a nomogram prediction model incorporating the FAB-M5 subtype, KIT mutation, and TP53 mutation was constructed, as illustrated in Figure 1.

### 3.5. Construction of the Scoring System

A scoring tool was developed based on the three variables identified in the final model (Table 3). 5 points were assigned for the presence of a KIT mutation, and 0 points for its absence. Similarly, 3 points were assigned for the presence of a TP53 mutation (0 for absence), and 2 points for the FAB-M5 subtype (0 for non-FAB-M5). The total score of the system ranged from 0 to 10, with the prediction score for VEN resistance calculated by summing the points from the three items. The scoring system was categorized into three groups: low risk (0–2 points), intermediate risk (3–5 points), and high risk (6–10 points). A statistically significant difference in resistance rates was observed among the three risk groups (32.4% vs. 75.2% vs. 97.4%, *p* < 0.001).

### 3.6. Survival Analysis

**Figure 2:**
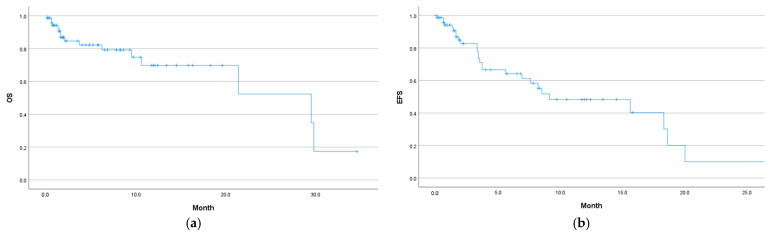
(**a**) Overall survival curve. (**b**) Event-free survival curve.

The median follow-up duration was 11.3 (4.5, 19.7) months. Objective responses were observed in a total of 74 patients, including 58 cases achieving a CRc + PR + MLFS, resulting in an ORR of 78.4%. Death occurred in 16 patients (21.6%), among whom 11 (68.8%) died due to relapse or disease progression, 7 (43.8%) due to severe infection, and 1 (6.3%) due to other causes. A total of 14 patients (23.3%) underwent allogeneic hematopoietic stem cell transplantation. The median OS was 29.5 months, with a 6-month OS rate of 82.2% and a 12-month OS rate of 69.8%. The median EFS was 9.1 months. The 6-month and 12-month EFS rates were 64.1% and 48.3%, respectively (Figure 2).

Multivariate Cox regression analysis indicated that the KIT gene (HR: 0.18, 95% CI: 0.04–0.87, *p* = 0.034) and the MAP3K14 gene (HR: 0.04, 95% CI: 0.01–0.35, *p* = 0.004) were independent protective factors for OS. Meanwhile, the MAP3K14 gene (HR: 0.05, 95% CI: 0.01–0.44, *p* = 0.007) was identified as an independent protective factor for EFS. No statistically significant differences in OS or EFS were observed among the other variable subgroups.

### 3.7. Incidence of VEN-Related Adverse Events

#### 3.7.1. Hematologic Adverse Events

A total of 68 patients (91.9%) experienced grade ≥ 3 hematologic AEs after treatment, including leukopenia in 58 cases (78.4%), neutropenia in 58 cases (78.4%), febrile neutropenia in 45 cases (60.8%), anemia in 56 cases (75.7%), and thrombocytopenia in 47 cases (63.5%).

#### 3.7.2. Infections

Among the 74 patients, 24 cases (32.4%) had CTCAE grade 3 infections, 15 cases (20.3%) had CTCAE grade 4 infections, and 7 cases (43.8%) died from infection. A total of 26 patients (35.1%) experienced at least one grade ≥ 3 infection.

A total of 91 infectious events were recorded, of which 45 events (49.5%) were grade ≥ 3. Among the grade ≥ 3 infections, 21 events (46.7%) were microbiologically confirmed, and 24 events (53.3%) were clinically defined. Among the recorded microbiologically confirmed infections, 12 events (38.7%) were bacterial, 10 events (32.3%) were viral, and 9 events (29.0%) were fungal. Invasive fungal infections occurred in 7 events, and no pneumocystis jirovecii pneumonia was observed.

Among the recorded infections, pneumonia occurred in 30 events (33.0%), respiratory tract infections in 25 events (27.5%), oral infections in 14 events (15.4%), bloodstream infections in 9 events (9.9%), gastrointestinal infections in 8 events (8.8%), and skin infections in 4 events (4.4%).

#### 3.7.3. Other Adverse Events

Gastrointestinal reactions were recorded in 12 patients (16.2%), including nausea/vomiting in 4 cases (33.3%), constipation in 1 case (8.3%), and diarrhea in 9 cases (75.0%). Other events included hypokalemia in 6 cases (8.1%), elevated transaminases in 3 cases (4.1%), tumor lysis syndrome in 2 cases (2.7%), rash in 4 cases (5.4%), and lower limb edema in 1 case (1.4%).

## 4. Discussion

As a novel BCL-2 inhibitor, VEN has largely transformed the treatment paradigm for elderly patients with newly diagnosed AML and those who are ineligible for high-intensity chemotherapy, while also providing more options for relapsed or refractory AML. In this study, we observed an ORR of 78.4%, which is higher than that reported in a study focusing on elderly patients with newly diagnosed AML (73%) [6] and the VIALE-A study (66.4%) [5]. The proportion of MDS-sAML (16.2%) and relapsed or refractory AML (10.8%) in this study was relatively low, and the differences in the distribution of AML subgroups among the enrolled patients compared to the aforementioned studies may be the primary reason for the higher ORR observed in this study.

This study analyzed patients with AML treated with VEN combined with HMAs, examining the influencing factors of primary resistance to VEN. The results indicated that the KIT gene (*p* = 0.012), TP53 gene (*p* = 0.010), and FAB-M5 subtype (*p* = 0.059) were associated with the occurrence of primary resistance to VEN combined with HMA therapy.

The drug resistance-related factors observed in this study corroborate previously reported mechanisms of VEN resistance. The primary resistance mechanisms of VEN-based combination therapy include the activation of kinase signaling pathways and biallelic TP53 perturbations. The expansion of TP53-mutated cells following VEN treatment suggests that this mutation directly mediates drug resistance and relapse risk [18]. The core mechanism lies in the fact that the TP53/BAX pathway is a critical node regulating drug sensitivity [19]. Mutations in TP53 lead to a shift in apoptotic dependency from BCL-2 to MCL-1 [20] and are accompanied by mitochondrial metabolic reprogramming [21]. Together, these findings elucidate the pathway through which TP53 mutations confer resistance to venetoclax. Additionally, studies have shown that simultaneous inhibition of BCL-2 and activation of p53 can produce a synthetic lethal effect in AML cells, highlighting the significant implications of TP53 mutation status when applying VEN-based therapies [22]. The kinase activation pathways involved are diverse, including KIT-ITD [18], supporting the conclusion of this study that TP53 and KIT gene mutations are associated with VEN resistance. Furthermore, this study found an association between the FAB-M5 subtype and VEN resistance. AML with a high degree of monocytic differentiation (e.g., FAB M4/M5) exhibits greater resistance to VEN. The underlying mechanism is that such cells rely less on BCL-2 and shift survival dependency to MCL-1, a shift supported by evidence of a BCL-2 expression gradient across FAB subtypes [23,24]. Of note, despite its non-significance, FAB-M5 was retained in the model due to its clinical relevance, allowing for a more comprehensive assessment.

In studies investigating factors influencing drug resistance, the FAB-M5 subtype, the presence of the RUNX1-RUNX1T1 fusion gene, FLT3-ITD mutation, and MDS-sAML have been associated with VEN primary resistance [25]. Our study did not observe an association between MDS-sAML and VEN resistance, which may be related to prior treatment regimens for MDS-sAML. Additionally, no correlation was found between VEN resistance and the FLT3 gene in our study, a factor warranting further investigation in subsequent research. Furthermore, this study explored the association between VEN blood concentration and the occurrence of resistance, a factor that, to date, has not been examined in similar studies within the existing literature [26,27].

Although individual biomarkers—namely KIT mutation, TP53 mutation, and the FAB-M5 subtype—have been independently validated in preclinical studies as being associated with resistance to venetoclax-based therapy, an integrated scoring system may offer additional utility for patient stratification in future clinical trials. First, given the frequent coexistence of multiple aberrations in AML patients, a cumulative score captures the aggregate resistance burden more comprehensively than binary classification based on any single marker. Second, conversion of these markers into an ordinal variable (low/intermediate/high) enables more refined randomization, ensuring balanced allocation of resistance severity across treatment arms. Third, this approach facilitates the identification of extreme phenotypic subgroups—particularly high-score patients—who may derive the greatest benefit from novel triplet regimens, whereas low-score patients could be spared unnecessarily intensive interventions.

We acknowledge several limitations in this study. The most significant is the limited sample size, which precludes further internal or external validation of the proposed scoring model. As a result, our findings should be interpreted as preliminary and exploratory, serving as a hypothesis-generating foundation rather than a ready-for-clinical-practice tool. Larger-scale studies with extended follow-up are necessary to confirm the reliability and clinical utility of our results. In subsequent research, we will actively enroll more patients to validate and potentially refine the model, aiming to integrate additional parameters to improve predictive accuracy.

This study offers preliminary and exploratory insights into the factors influencing VEN resistance in AML patients, which necessitate confirmation in larger-scale cohorts. The complexity of polyclonal resistance presents significant clinical challenges for salvage therapy, particularly in relapsed or refractory hematologic malignancies where multiple subclones with distinct resistance mechanisms coexist. This heterogeneity often renders single-agent or conventional combination regimens ineffective. Integrating molecular characteristics with clinical phenotypes to guide drug selection can provide a crucial foundation for developing individualized treatment strategies, enabling clinicians to prioritize mutations driving clonal expansion. For patients carrying targetable gene mutations, triple therapy combining VEN with hypomethylating agents and corresponding targeted drugs may represent a promising therapeutic approach.

## 5. Conclusions

This study identified KIT mutation, TP53 mutation, and FAB-M5 subtype as independent factors associated with primary resistance to VEN combined with HMAs in AML patients. Based on these variables, a nomogram and risk scoring system (0–2 low, 3–5 intermediate, 6–10 high) were constructed, showing significant differences in non-response rates across risk groups (*p* < 0.001). This integrated scoring system may aid patient stratification in future studies. However, the small sample size precludes validation, so findings are preliminary. Larger studies are needed to confirm the model’s utility for guiding individualized treatment.

## Figures and Tables

**Table 1:** Patients’ characteristics.

Characteristics	CR, CRi, PR, n (%)	NR, n (%)	*p*
Sex, n			0.311
Male	34 (56.7)	10 (71.4)	
female	26 (43.3)	4 (28.6)	
Age, Mean ± SD	64.13 ± 13.90	64.36 ± 9.47	0.955
R/R, n	8 (13.3)	4 (28.6)	0.322
MDS-sAML, n	5 (8.3)	3 (21.4)	0.346
FAB-M5, n	5 (8.3)	4 (28.6)	0.103
2022 ELN risk, n			0.934
1	10 (16.7)	2 (14.3)	
2	11 (18.3)	3 (21.4)	
3	33 (55.0)	9 (64.3)	
Genetic Features, n			
ASXL1	10 (16.7)	2 (14.3)	0.999
CEBPA	3 (5.0)	1 (7.1)	0.576
DNMT3A	12 (20.0)	1 (7.1)	0.454
FLT3-ITD	15 (25.0)	2 (14.3)	0.613
KIT	1 (1.7)	3 (21.4)	0.020
KRAS	4 (6.7)	1 (7.1)	0.999
NPM1	10 (16.7)	2 (14.3)	0.999
NRAS	7 (11.7)	1 (7.1)	0.990
PTPN11	3 (5.0)	1 (7.1)	0.576
RUNX1	4 (6.7)	2 (14.3)	0.692
TET2	3 (5.0)	3 (21.4)	0.138
TP53	4 (6.7)	4 (28.6)	0.058
WT1	6 (10.0)	2 (14.3)	0.999
SRSF2	4 (6.7)	2 (14.3)	0.692
Complex Karyotype	2 (3.3)	1 (7.1)	0.472
MLL-rearrangement	4 (6.7)	1 (7.1)	0.999
RUNX1-RUNX1T1	3 (5.0)	1 (7.1)	0.576

**Table 2:** Univariate and multivariate logistic regression analysis.

Variables	Univariate Analysis			Multivariate Analysis		
	OR	95% CI	*p*	OR	95% CI	*p*
FAB-M5	4.40	1.00–19.28	0.049	5.10	0.94–27.73	0.059
KIT	16.09	1.53–169.22	0.021	24.02	2.02–285.53	0.012
TET2	5.18	0.92–29.10	0.062			
TP53	5.60	1.20–26.14	0.028	8.90	1.68–47.16	0.010

**Table 3:** Scoring System for VEN Resistance.

Variables	Estimate	Point
KIT		
No		0
Yes	3.179	5
TP53		
No		0
Yes	2.186	3
FAB-M5		
No		0
Yes	1.629	2
Total		10

## Data Availability

Data available on request due to restrictions. The data presented in this study are available on request from the corresponding author due to ethical reasons.

## References

[1] DiNardo C.D., Jonas B.A., Pullarkat V., Thirman M.J., Garcia J.S., Wei A.H., Konopleva M., Döhner H., Letai A., Fenaux P. (2020). Azacitidine and Venetoclax in Previously Untreated Acute Myeloid Leukemia. N. Engl. J. Med..

[2] Pratz K.W., Jonas B.A., Pullarkat V., Thirman M., Garciaz S., Döhner H., Récher C., Fiedler W., Yamamoto K., Wang J. (2024). Long-Term Follow-Up of VIALE-A: Venetoclax and Azacitidine in Chemotherapy-Ineligible Untreated Acute Myeloid Leukemia. Am. J. Hematol..

[3] Vachhani P., Flahavan E.M., Xu T., Ma E., Montez M., Gershon A., Onishi M., Jin H., Ku G., Flores B. (2022). Venetoclax and Hypomethylating Agents as First-line Treatment in Newly Diagnosed Patients with AML in a Predominately Community Setting in the US. Oncologist.

[4] Sanz-Solas A., Saiz-Rodríguez M., Simal S.C., Rodríguez-Veiga R., Solana-Altabella A., Montesinos P., Labrador J. (2025). Comparative efficacy of venetoclax and hypomethylating agents in acute myeloid leukemia treatment: A meta-analysis of clinical trials and Real-World outcomes. Ann. Hematol..

[5] Othman J., Lam H.P.J., Leong S., Basheer F., Abdallah I., Fleming K., Mehta P., Yassin H., Laurie J., Austin M. (2024). Real-world outcomes of newly diagnosed AML treated with venetoclax and azacitidine or low-dose cytarabine in the UK NHS. Blood Neoplasia.

[6] Wurm S., Moritz J.M., Petzer V., Wolf D., Gleixner K.V., Sperr W.R., Groiss C., Machherndl-Spandl S., Eisenwort G., Koller E. (2025). Efficacy assessment following shortened venetoclax exposure in AML patients treated with venetoclax plus hypomethylating agents: A real-world, multicentric study. Blood Cancer J..

[7] Roth J.A., D’Amico P.G., Cappelleri J.C., Donckels E.A., Yu A., Okunev I., Petrilla A., Purcell S., Ma J., Russell A. (2025). Real-world treatment and utilization of venetoclax for incident acute myeloid leukemia in a Medicare population. Future Oncol..

[8] Chin K.K., Derkach A., Famulare C., Gupta G.K., Borge P.D., Geyer M.B., Goldberg A.D., Haque T., Park J.H., Roeker L.E. (2025). HMA/VEN treatment modifications and associated outcomes in IDH-mutant AML. Leuk. Lymphoma.

[9] Shimony S., Stahl M., Stone R.M. (2025). Acute Myeloid Leukemia: 2025 Update on Diagnosis, Risk-Stratification, and Management. Am. J. Hematol..

[10] Nwosu G.O., Ross D.M., Powell J.A., Pitson S.M. (2024). Venetoclax therapy and emerging resistance mechanisms in acute myeloid leukaemia. Cell Death Dis..

[11] Chatzilygeroudi T., Karantanos T., Pappa V. (2025). Unraveling Venetoclax Resistance: Navigating the Future of HMA/Venetoclax-Refractory AML in the Molecular Era. Cancers.

[12] Diamantidis M.D. (2025). Factors affecting response and resistance to venetoclax in acute myeloid leukemia. Front. Oncol..

[13] Lu H., Wang H., Wang Q., Huang D., Han Y., Wang H., Jiang P., Qian X., Mao L., Yang M. (2025). Dynamic evolution of venetoclax resistance in acute myeloid leukemia unveiled by longitudinal single-cell RNA-seq. Cancer Lett..

[14] Jin P., Wang D., Shen J., Jin Q., Zhang H., Liu X., He M., Jin W., Li Y., Dong F. (2025). Precision prediction of venetoclax-azacitidine treatment efficacy in acute myeloid leukemia via integrative drug screening and machine learning. Cell Rep. Med..

[15] Trac Q.T., Pawitan Y., Mou T., Erkers T., Östling P., Bohlin A., Österroos A., Vesterlund M., Jafari R., Siavelis I. (2023). Prediction model for drug response of acute myeloid leukemia patients. npj Precis. Oncol..

[16] Zhang Y., Martinez J., Tercan B., Kuusanmäki H., Emmert-Streib F., Chandraseelan J.G., Farea A., Yli-Harja O., Heckman C.A., Gibbs D.L. (2025). Digital twin models for predicting venetoclax and azacitidine-induced neutropenia in patients with acute myeloid leukemia. npj Digit. Med..

[17] Dhakal P., Bates M., Tomasson M.H., Sutamtewagul G., Dupuy A., Bhatt V.R. (2023). Acute myeloid leukemia resistant to venetoclax-based therapy: What does the future hold?. Blood Rev..

[18] Dinardo C.D., Pratz K., Pullarkat V., Jonas B.A., Arellano M., Becker P.S., Frankfurt O., Konopleva M., Wei A.H., Kantarjian H.M. (2019). Venetoclax combined with decitabine or azacitidine in treatment-naive, elderly patients with acute myeloid leukemia. Blood.

[19] DiNardo C.D., Tiong I.S., Quaglieri A., MacRaild S., Loghavi S., Brown F.C., Thijssen R., Pomilio G., Ivey A., Salmon J.M. (2020). Molecular patterns of response and treatment failure after frontline venetoclax combinations in older patients with AML. Blood.

[20] Nechiporuk T., Kurtz S.E., Nikolova O., Liu T., Jones C.L., D’Alessandro A., Culp-Hill R., D’Almeida A., Joshi S.K., Rosenberg M. (2019). The TP53 Apoptotic Network Is a Primary Mediator of Resistance to BCL2 Inhibition in AML Cells. Cancer Discov..

[21] Michels J., Johnson P.W., Packham G. (2005). Mcl-1. Int. J. Biochem. Cell Biol..

[22] Pan R., Ruvolo V., Mu H., Leverson J.D., Nichols G., Reed J.C., Konopleva M., Andreeff M. (2017). Synthetic Lethality of Combined Bcl-2 Inhibition and p53 Activation in AML: Mechanisms and Superior Antileukemic Efficacy. Cancer Cell.

[23] Pei S., Pollyea D.A., Gustafson A., Stevens B.M., Minhajuddin M., Fu R., Riemondy K.A., Gillen A.E., Sheridan R.M., Kim J. (2020). Monocytic Subclones Confer Resistance to Venetoclax-Based Therapy in Patients with Acute Myeloid Leukemia. Cancer Discov..

[24] Zhang H., Nakauchi Y., Köhnke T., Stafford M., Bottomly D., Thomas R., Wilmot B., McWeeney S.K., Majeti R., Tyner J.W. (2020). Integrated analysis of patient samples identifies biomarkers for venetoclax efficacy and combination strategies in acute myeloid leukemia. Nat. Cancer.

[25] Zong L., Yin M., Kong J., Zhang J., Song B., Zhu J., Xue S., Wu X., Wu D., Bao X. (2023). Development of a scoring system for predicting primary resistance to venetoclax plus hypomethylating agents (HMAs) in acute myeloid leukemia patients. Mol. Carcinog..

[26] Li Y., Wan Q., Wan J., Xiao X., Hu J., Yang X., Kong F., Wang J., Song B., Li Z. (2024). Plasma concentrations of venetoclax and Pharmacogenetics correlated with drug efficacy in treatment naive leukemia patients: A retrospective study. Pharmacogenom. J..

[27] Yang X., Mei C., He X., He L., Lu X., Tong H., Lou Y. (2022). Quantification of Venetoclax for Therapeutic Drug Monitoring in Chinese Acute Myeloid Leukemia Patients by a Validated UPLC-MS/MS Method. Molecules.

